# Disruption of the ER-α36-EGFR/HER2 Positive Regulatory Loops Restores Tamoxifen Sensitivity in Tamoxifen Resistance Breast Cancer Cells

**DOI:** 10.1371/journal.pone.0107369

**Published:** 2014-09-09

**Authors:** Li Yin, Xin-Tian Zhang, Xiu-Wu Bian, Yu-Ming Guo, Zhao-Yi Wang

**Affiliations:** 1 Departments of Medical Microbiology and Immunology, Creighton University Medical School, Omaha, Nebraska, United States of America; 2 Institute of Pathology and Southwest Cancer Center, Southwest Hospital, Third Military Medical University, Chongqing, China; University of South Alabama, United States of America

## Abstract

Tamoxifen provided a successful treatment for ER-positive breast cancer for many years. However, most breast tumors develop tamoxifen resistance and are eventually refractory to tamoxifen therapy. The molecular mechanisms underlying development of tamoxifen resistance have not been well established. Recently, we reported that breast cancer cells with high levels of ER-α36, a variant of ER-α, were resistant to tamoxifen and knockdown of ER-α36 expression in tamoxifen resistant cells with the shRNA method restored tamoxifen sensitivity, indicating that gained ER-α36 expression is one of the underlying mechanisms of tamoxifen resistance. Here, we found that tamoxifen induced expression of ER-α36-EGFR/HER2 positive regulatory loops and tamoxifen resistant MCF7 cells (MCF7/TAM) expressed enhanced levels of the loops. Disruption of the ER-α36-EGFR/HER2 positive regulatory loops with the dual tyrosine kinase inhibitor Lapatinib or ER-α36 down-regulator Broussoflavonol B in tamoxifen resistant MCF7 cells restored tamoxifen sensitivity. In addition, we also found both Lapatinib and Broussoflavonol B increased the growth inhibitory activity of tamoxifen in tumorsphere cells derived from MCF7/TAM cells. Our results thus demonstrated that elevated expression of the ER-α36-EGFR/HER2 loops is one of the mechanisms by which ER-positive breast cancer cells escape tamoxifen therapy. Our results thus provided a rational to develop novel therapeutic approaches for tamoxifen resistant patients by targeting the ER-α36-EGFR/HER2 loops.

## Introduction

Endocrine therapy using antiestrogen tamoxifen (TAM) is currently the most effective treatment for advanced ER-positive breast cancer. Tamoxifen acts through ER pathway, which has been proven to reduce relapse, death rates and risk of contralateral breast cancer. However, patients often develop resistance to tamoxifen, which limit its effectiveness [1]–[4]. Many researches were conducted to understand the molecular pathways involved in tamoxifen resistance and have revealed that multiple signaling molecules and pathways such as EGFR and HER2 [5], [6]. All these pathways often bypass the requirement of estrogen signaling for growth of ER-positive breast cancer cells.

Both experimental and clinical evidence have indicated that the HER2 (Human epidermal growth factor receptor 2) and EGFR (Epidermal growth factor receptor) signaling pathways interact with the estrogen-signaling pathway. Experimental evidence has shown that estrogen-dependent MCF7 cells that over-express HER2 are rendered tamoxifen resistant [5], [6]. Hence the HER2 pathway has been investigated for its contribution towards development of tamoxifen resistance and now HER2 has been proposed as a potential marker of tamoxifen sensitivity. Many clinical studies have found an association between HER2 overexpression and tamoxifen failure [7]–[15]. Thus, the combination therapy by targeting both HER2 and ER-α was hypothesized and tested in preclinical studies [16]–[18]. Chu et al., reported that the dual kinase inhibitor Laptinib for HER2 and EGFR cooperates with tamoxifen to inhibit cell proliferation in antiestrogen resistant breast cancer [19].

Previously, our laboratory identified and cloned a variant of ER-α, ER-α36, which has a molecular weight of 36-kDa [20], [21]. The transcript of ER-α36 is initiated from a previously unidentified promoter in the first intron of the ER-α gene [22]. This ER-α differs from the original 66 kDa ER-α (ER-α66) because it lacks both transcriptional activation domains (AF-1 and AF-2) but retains the DNA-binding and dimerization domains, and partial ligand-binding domain [20]. ER-α36 is mainly expressed at the plasma membrane and mediates membrane-initiated estrogen signaling [21]. Previously, We reported that the breast cancer patients with tumors expressing high levels of ER-α36 less benefited from TAM therapy than those with low levels of ER-α36 expression and ER-α36 expression is well correlated with HER2 expression [23], suggesting that gained ER-α36/HER2 expression is one of the underlying mechanisms of TAM resistance. Indeed, ER-α36 is able to mediate agonist activity of TAM such as activation of the MAPK (mitogen-activated protein kinase)/ERK (extracellular regulated protein kinases) and the PI3K (Phosphoinositides 3-kinase)/AKT signaling pathways [24], [25] and is involved in development of TAM resistance [26], [27]. Recently, we reported the existence of positive regulatory loops between ER-α36 and EGFR/HER2 in ER-negative breast cancer cells [28], [29]. In triple-negative breast cancer MDA-MB-231 and MDA-MB-436 cells, knockdown of ER-α36 expression enhances EGFR protein degradation through the proteasome system while EGFR signaling pathway up-regulates the promoter activity of ER-α36 through an Ap1 binding site in the 5′ flanking sequence of ER-α36 gene [28]. In HER2 overexpressing breast cancer SKBR3 cells, ER-α36-mediated signaling positively regulates HER2 transcription while HER2 signaling up-regulates the promoter activity of ER-α36. However, the function and underlying mechanisms of these regulatory loops in development of TAM resistance of ER-positive breast cancer cells are largely unknown,

Here, we sought to examine whether the ER-α36-EGFR/HER2 positive regulatory loops also exist in ER-positive breast cancer cells and whether these loops are involved in development of tamoxifen resistance. We also to tested the possibility of disruption of these loops with chemicals to restore TAM sensitivity in TAM resistant cells. Using TAM sensitive breast cancer MCF7 cells and TAM resistant MCF7 cells as models, we investigated the function of the ER-α36-EGFR/HER2 positive regulatory loops in TAM resistance.

## Methods

### Chemicals and antibodies

Tamoxifen was purchased from Sigma Chemical Co. (St. Louis, MO). Broussoflavonol B was obtained from Shenogen Pharma Group (Beijing, P.R. China). Anti-phospho-EGFR (Tyr1045) and –HER2/ErbB2 (Tyr1221/1222) as well as anti-EGFR and -HER2/ErbB2 (D8F12) antibodies were purchased from Cell Signaling Technology (Boston, MA). Antibodies of ER-α66 and β-actin were purchased from Santa Cruz Biotechnology (Santa Cruz, CA). Polyclonal anti-ER-α36 antibody was generated and characterized as described before [26].

### Cell culture and establishment of stable cell lines

MCF7 and T47D cells were obtained from ATCC (American Type Culture Collection, Manassas, VA). H3396 cells were kindly provided by Dr. Leia Smith at the Seattle Genetics Inc. MCF7/ER36, MCF7/Si36, MCF7/TAM and MCF7/TAM/Si36 cells were established as described before [28], [30]. All cells were maintained at 37°C in a humidified atmosphere containing 10% CO2 in IMEM media without phenol red and 10% fetal calf serum.

To examine cell growth in the presence or absence of TAM as well as other chemicals, cells maintained for three days in phenol red-free DMEM plus 2.5% dextran-charcoal-stripped fetal calf serum (HyClone, Logan, UT) were treated with different concentrations of TAM and other chemicals, or vehicle as a control. The cells were seeded at 1×10^4^ cells per dish in 60 mm dishes and the cell numbers were determined using the ADAM automatic cell counter (Digital Bio., Korea) after seven days. Five dishes were used for each treatment and experiments were repeated more than three times.

### Western blot analysis

For immunoblot analysis, cells washed with PBS were lysed with the lysis buffer (50 mM Tris-HCl pH 8.0, 150 mM NaCl, 0.25 mM EDTA pH 8.0, 0.1% SDS, 1% Triton X-100, 50 mM NaF) supplemented with protease and phosphatase inhibitors (Sigma). The protein amounts were measured using the DC protein assay kit (BIO-RAD Laboratories, Hercules, CA). The same amounts of the cell lysates were boiled for five minutes in loading buffer and separated on a SDS-PAGE gel. After electrophoresis, the proteins were transferred to a PVDF membrane. The membranes were probed with various primary antibodies, HRP-conjugated secondary antibodies, and visualized with enhanced chemiluminescence (ECL) detection reagents (GE Healthcare Bio-Sciences Corp., Piscataway, NJ). All Western blot assays were repeated three times.

### Tumorsphere formation and flow cytometry analysis

To establish tumorspheres, cells were seeded onto Corning Ultra-Low Attachment 6-well plate (Corning Incorporated, CA) at 10,000 cells/ml and cultured seven days in the tumorsphere medium: phenol-red free DMEM/F12 medium (Invitrogen) supplemented with 1× B-27 (Invitrogen), 20 ng/ml epidermal growth factor (Sigma-Aldrich) and 20 ng/ml basic fibroblast growth factor (ProSpec, NJ), 0.5 µg/mL hydrocortisone (Sigma). Tumorspheres were collected, washed with PBS, and incubated with Trypsin-EDTA (0.25%/0.5 mM) for two minutes at 37°C to dissociated cells. The number of tumorspheres and dissociated cells were counted using a Multisizer 3 Coulter Counter (Beckman Coulter, Brea, CA) and the ADAM automatic cell counter, respectively. For TAM treatment assays, tumorspheres were treated with tamoxifen or vehicle (ethanol) as a control. Three dishes were used for each group and all experiments were repeated three times.

To assess the effects of disruption of the positive-regulatory loops on the self-renewal of the stem-like cells, tumorspheres were dissociated and cell number was determined. The cells from 1^st^ generation of tumorspheres were seeded onto Ultra-Low Attachment 6-well plate at 5,000 cells/ml and cultured five days in the presence or absence of different chemicals to form 2^nd^ generation tumorspheres. The number of tumorspheres and dissociated cells were counted using a Multisizer 3 Coulter Counter. Three dishes were used for each group and all experiments were repeated three times.

For CD44^+^/CD24^−^ cell analysis, single cell suspension washed with cold PBS/1% BSA were incubated with PerCP-Cy5.5 mouse anti-human CD44 and PE mouse anti-human CD24 in PBS/1% BSA for 30 minutes at 4°C. After incubation, the cells were washed twice in cold PBS/1% BSA and re-suspended in cold PBS/1% BSA for flow cytometry analysis.

### Statistical analysis

Data from at least three independent experiments are expressed as the mean ± standard error (SE) using the GraphPad InStat software program. Each data point of cell proliferation and Tumorsphere formation was run at least in triplicates and independent experiments were performed at least three times, Tukey-Kramer Multiple Comparisons Test was also used, and the significance was accepted for *P*<0.05.

## Results

### Tamoxifen induces ER-α36, EGFR and HER2 expression in ER-positive breast cancer MCF7 cells

**Figure 1:**
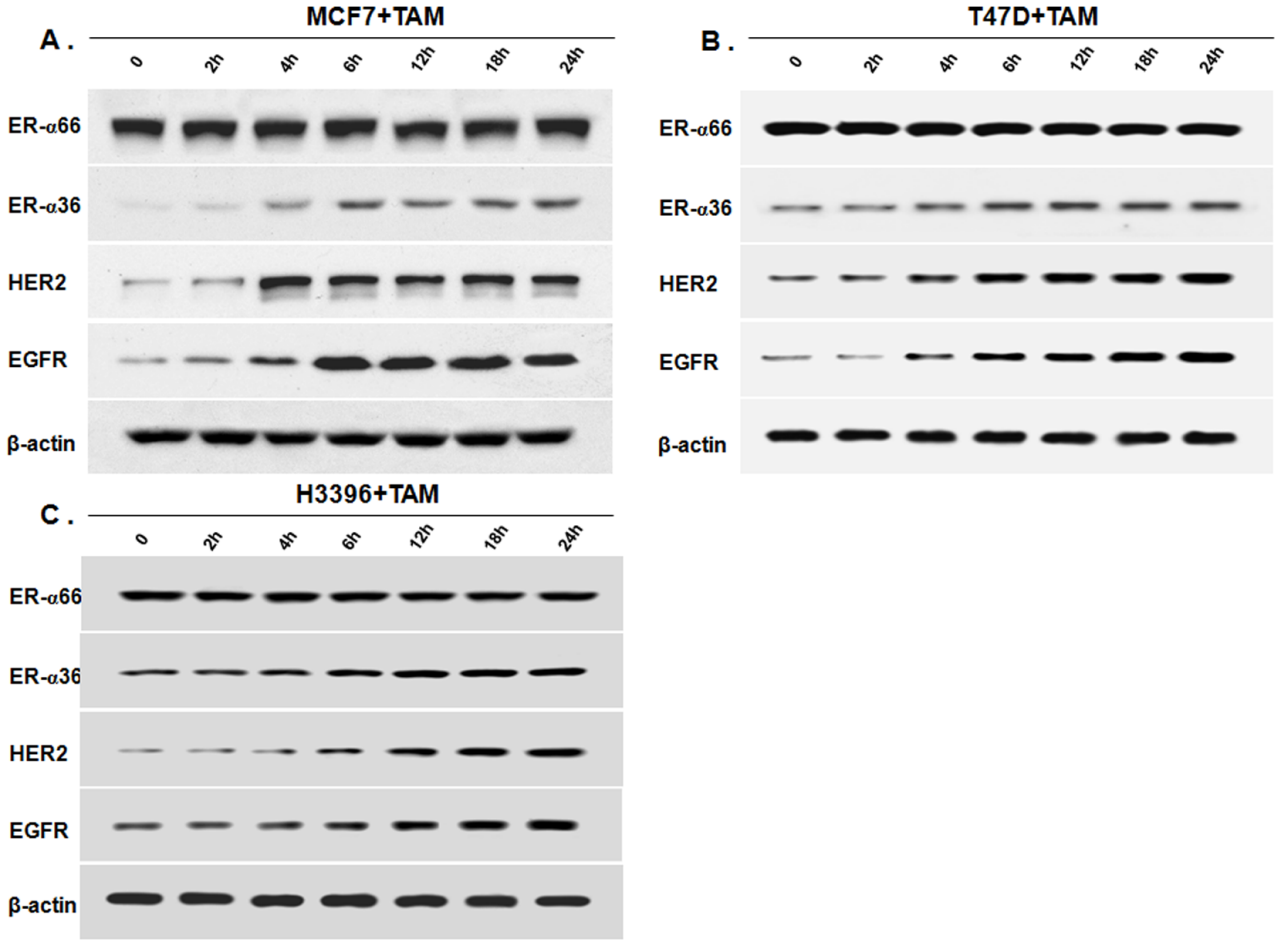
Tamoxifen induces ER-α36, HER2 and EGFR expression. Western blot analysis of the expression levels of ER-α66, ER-α36, HER2 and EGFR in ER-positive breast cancer MCF7 (A), T47D (B) and H3396 (C) cells treated with 1 µM of tamoxifen (TAM) for indicated time period.

Previously, our laboratory identified and cloned a 36 kDa variant of ER-α, ER-α36 that functions differently from the 66 kDa full-length ER-α, ER-α66 [21], [22]. Recently, we found that there exist ER-α36-EGFR/HER2 positive regulatory loops in ER-negative breast cancer cells [21. 22]. We then decided to examine whether the same regulatory loops are also in ER-positive breast cancer cells and involved in development of TAM resistance. The steady state levels of ER-α36, EGFR and HER2 in ER-positive breast cancer MCF7 cells treated with 1 µM of TAM for different time periods were examined with Western blot analysis. After TAM treatment, the levels of ER-α36, EGFR and HER2 expression were dramatically increased in MCF7 cells (Figure 1A), consistent with the previous reports that tamoxifen treatment induced ER-α36 in MCF7 cells [26], [27] and EGFR/HER2 expression in MCF7 xenograft tumors [31]. The same results were also observed in ER-positive breast cancer T47D and H3396 cells (Figure 1B & C).

### Tamoxifen induces the expression of ER-α36, EGFR and HER2 through the positive regulatory loops in MCF7 cells

**Figure 2:**
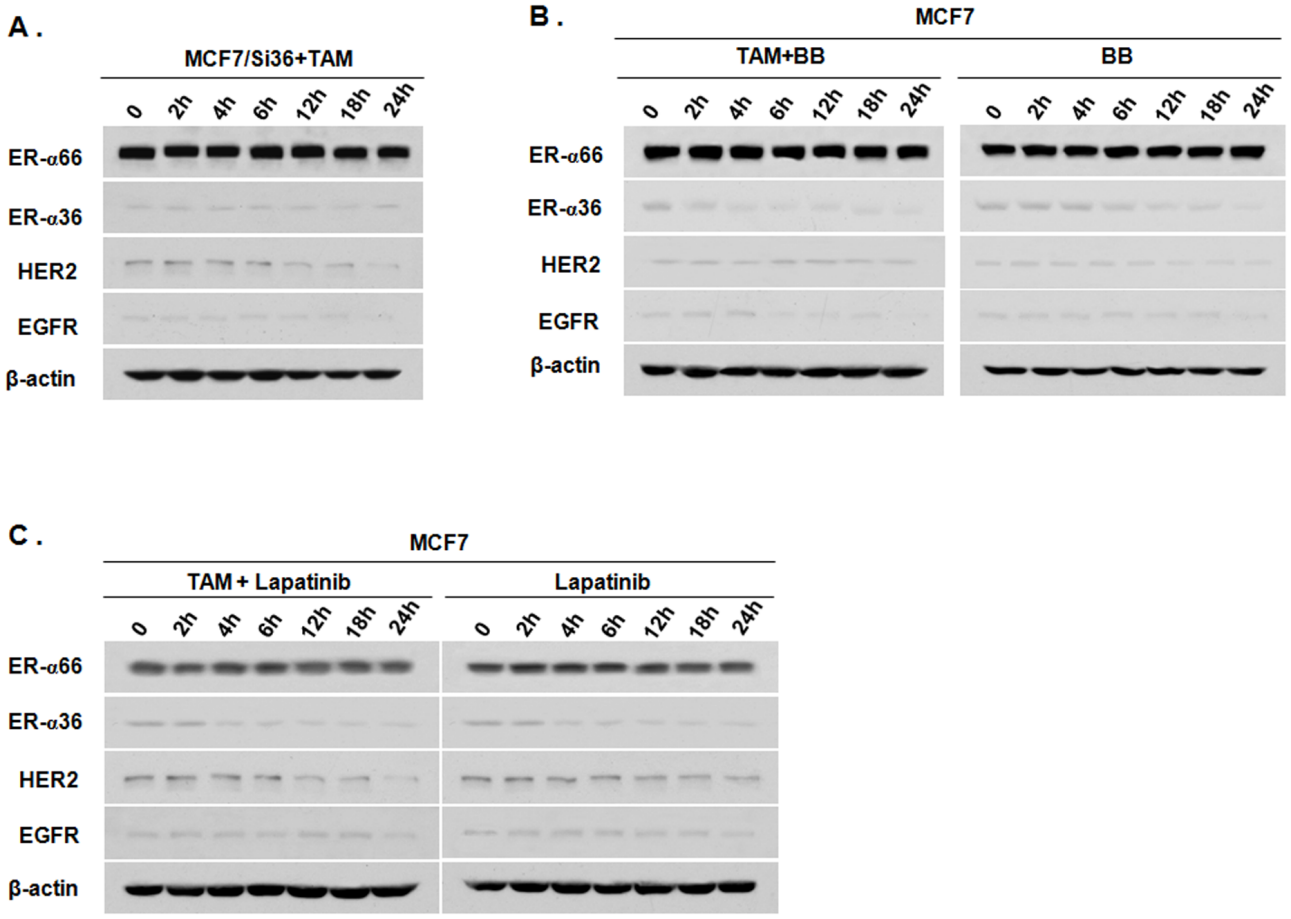
Tamoxifen induces ER-α36, HER2 and EGFR expression via the ER-α36-EGFR/HER2 positive regulatory loops. A. Western blot analysis of the expression levels of ER-α66, ER-α36, HER2 and EGFR in MCF7 cells with knocked-down levels of ER-α36 expression (MCF7/Si36) treated with 1 µM of TAM for indicated time period. B. ER-α66, ER-α36, HER2 and EGFR expression in MCF7 cells treated with 1 µM of TAM together with 1 µM of Broussoflavonol B (BB) for indicated time period. C. Western blot analysis of ER-α66, ER-α36, HER2 and EGFR in MCF7 cells treated with 1 µM of TAM and 1 µM of Lapatinib for indicated time period.

To examine if the ER-α36-EGFR/HER2 positive regulatory loops are involved in induction of ER-α36, EGFR and HER2 by TAM, we used a cell line MCF7/Si36; MCF7 cells that express knocked-down levels of ER-α36 [30]. TAM (1 µM) treatment failed to induce ER-α36, EGFR and HER2 expression in this cell line (Figure 2A), indicating that ER-α36 is involved in the induction of EGFR and HER2 by TAM. To confirm this result, we also treated the cells with Broussoflavonol B (BB), an ER-α36 downregulator [32], [33] together with TAM. Western blot analysis revealed that BB blocked the induction of ER-α36 as well as EGFR and HER2 by TAM (Figure 2B). Our results thus indicated that ER-α36 is involved in the induction of EGFR and HER2 expression by TAM.

To examine whether the signaling pathways of EGFR and HER2 contribute to the induction of ER-α36 expression by TAM, we treated MCF7 cells with the dual kinase inhibitor Lapatinib together with TAM and found that the EGFR/HER2 dual inhibitor blocked TAM induction of ER-α36 (Figure 2C). Taken together, our results demonstrated that TAM treatment induced expression of ER-α36, EGFR and HER2 presumably through the ER-α36-EGFR/HER2 positive regulatory loops.

### Tamoxifen resistant MCF7 cells exhibit enhanced expression of ER-α36-EGFR/HER2 loops

**Figure 3:**
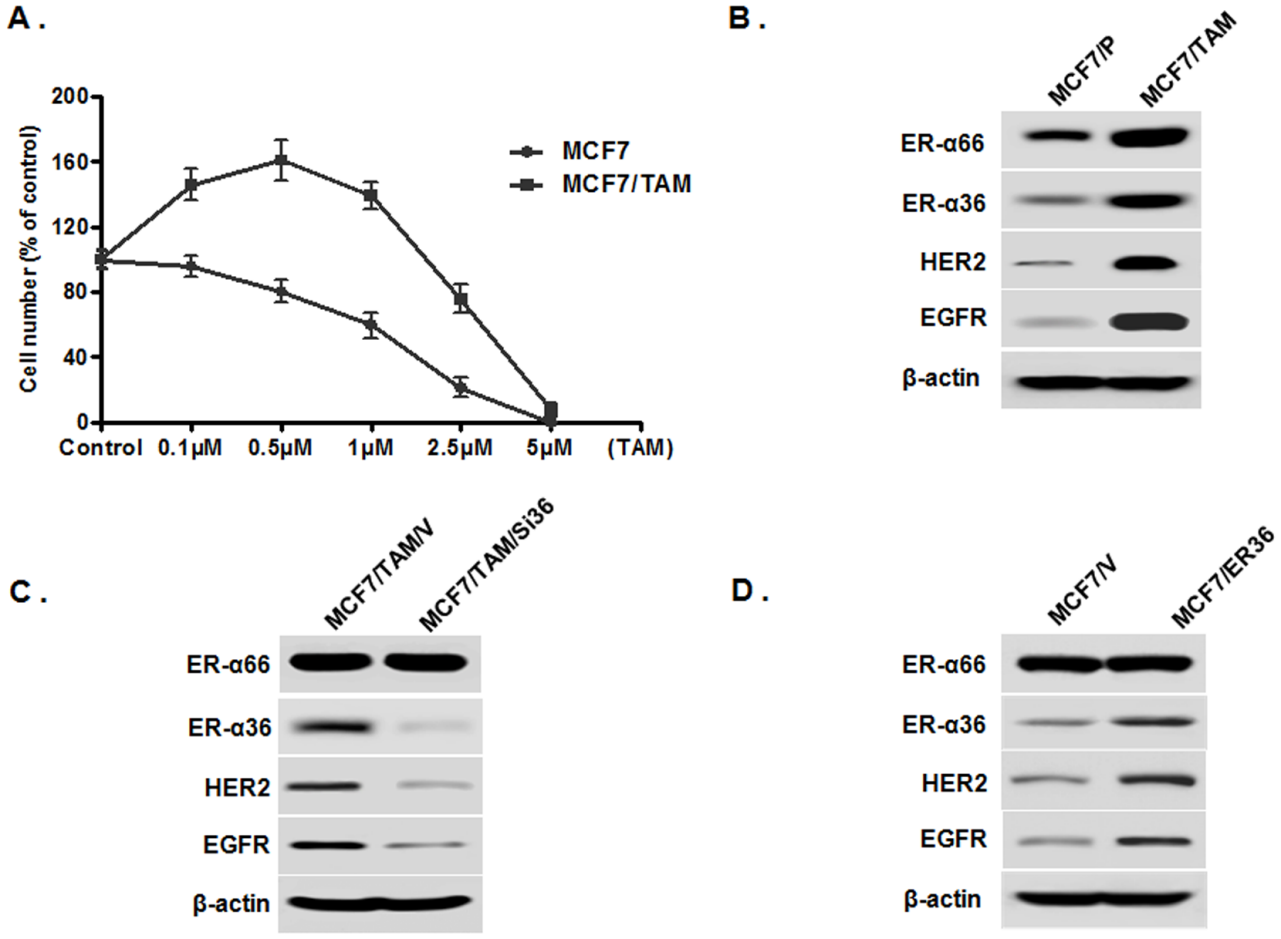
Tamoxifen resistant ER-positive breast cancer MCF7 cells express ER-α36-EGFR/HER2 regulatory loops. A. ER-positive breast cancer MCF7 cells and tamoxifen resistant MCF7 cells (MCF7/TAM) cells were treated with indicated concentrations of TAM for seven days and survived cells were counted. Each point represents the means of three experiments; bars, SE. B. Western blot analysis of the expression levels of ER-α66, ER-α36, EGFR and HER2 in parental MCF7 (MCF7/P) and MCF7/TAM cells. C. Expression of ER-α66, ER-α36, EGFR and HER2 in MCF7/TAM cells transfected with the empty expression vector (MCF7/TAM/V) and MCF7/TAM cells with knocked-down levels of ER-α36 expression (MCF7/TAM/Si36). D. Expression of ER-α66, ER-α36, EGFR and HER2 in MCF7 cells transfected with the empty expression vector (MCF7/V) and MCF7 cells with forced expression of ER-α36 recombinant DNA (MCF7/ER36).

Recently, we reported establishment of a TAM resistant MCF7 cell line (MCF7/TAM) by continuous treatment of TAM sensitive MCF7 cells with 1 µM of TAM for six months [28]. This cell line exhibited resistance to TAM treatment compared to the parental cells and lower concentrations of TAM (≤1 µM) even acted as an agonist in MCF7/TAM cells (Figure 3A). Western blot analysis revealed that ER-α36, EGFR and HER2 were all expressed at higher levels in MCF7/TAM cells compared to the MCF7 parental cells (Figure 3B), suggesting that MCF7 cells gained expression of the ER-α36-EGFR/HER2 regulatory loops during development of acquired TAM resistance.

To confirm that gained expression of ER-α36 is involved in elevated expression of EGFR and HER2, we used a cell line that expressed knocked-down levels of ER-α36 (MCF7/TAM/Si36) [26]. Western blot analysis revealed that both EGFR and HER2 expression was dramatically down-regulated in MCF7/TAM/Si36 cells compared to the control cells transfected with the empty expression vector (Figure 3C). Our data thus suggested that elevated ER-α36 expression is involved in enhanced expression of EGFR and HER2 in TAM resistant breast cancer cells.

To further confirm that enhanced levels of ER-α36 expression contribute to increased expression of both EGFR and HER2, we also used a stable cell line MCF7/ER36 that expresses high levels of recombinant ER-α36. Western blot analysis demonstrated that recombinant ER-α36 was highly expressed in MCF7/ER36 cells compared to the control MCF7 cells transfected with the empty expression vector (Figure 3D). Both EGFR and HER2 were also highly expressed in MCF7/ER36 cells (Figure 3D), indicating that increased ER-α36 expression is one of the mechanisms by which ER-positive breast cancer cells gained EGFR and HER2 expression.

### Dual kinase inhibitor Lapatinib downregulates ER-α36 expression and sensitizes MCF7/TAM cells to tamoxifen

**Figure 4:**
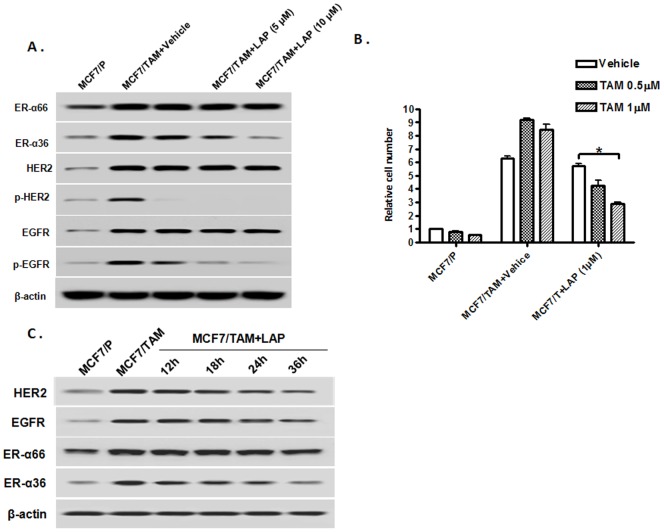
Dual kinase inhibitor Lapatinib downregulates ER-α36 expression and sensitizes MCF7/TAM cells to tamoxifen. A. Western blot analysis of the expression of ER-α36 and 66 as well as EGFR and HER2, and levels of EGFR and HER2 phosphorylation in parental MCF7 (MCF7/P) and MCF7/TAM cells treated with indicated concentrations of Lapatinib for 12 hours. B. Cells were treated with indicated concentrations of TAM together with vehicle or 1 µM of Lapatinib (LAP) for seven days and the numbers of survived cells were counted. The columns represent the means of three experiments; bars, SE. *, P<0.05 for MCF/TAM cells treated with vehicle vs cells treated with 1 µM of tamoxifen and Lapatinib.

Previously, we reported that increased level of ER-α36 expression is one of the underlying mechanisms of TAM resistance and knockdown of ER-α36 expression restored TAM sensitivity in MCF7/TAM cells [26]. Here, we sought to examine whether disruption of the ER-α36 and EGFR/HER2 regulatory loops using chemical inhibitors restores TAM sensitivity in TAM resistant MCF7 cells. We first treated MCF7/TAM cells with different concentrations of Lapatinib for 12 hours and the level of ER-α36 expression was examined with Western blot analysis. We found that Lapatinib inhibited phosphorylation of both EGFR and HER2 effectively and also downregulated ER-α36 expression in MCF7/TAM cells (Figure 4A). Lapatinib treatment significantly increased sensitivity to TAM in MCF7/TAM cells (Figure 4B). However, we did not find significant changes of the levels of EGFR and HER2 expression in cells treated with Lapatinib for 12 hours, which seems contradictory to the model of the ER-α36-EGFR/HER2 signaling loops. When we increased Lapatinib treatment to a longer period of time, we observed the expression levels of EGFR and HER2 were dramatically downregulated in the cells treated for 36 hours (Figure 4C). Taken together, these results demonstrated that the dual kinase inhibitor Lapatinib was able to disrupt the ER-α36-EGFR/HER2 positive regulatory loops and restored TAM sensitivity in TAM resistant cells.

### ER-α36 downregulator Broussoflavonol B also diminishes EGFR/HER2 expression and restores TAM sensitivity

**Figure 5:**
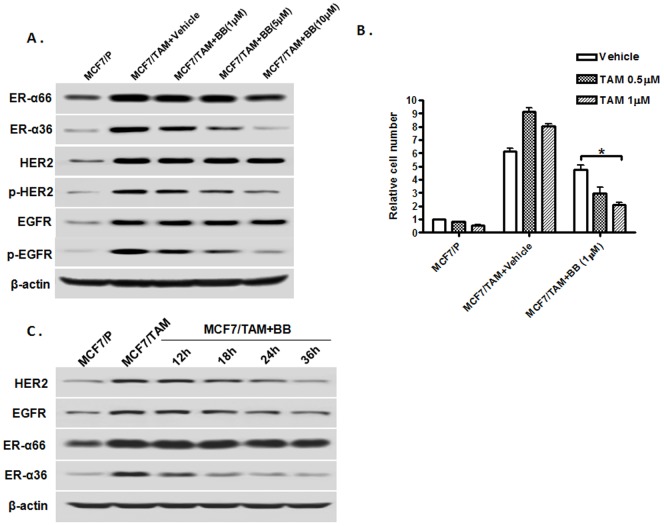
ER-α36 disruptor Broussoflavonol B restores TAM sensitivity. A. Western blot analysis of the expression of ER-α36, ER-α66, EGFR and HER2, and levels of EGFR and HER2 phosphorylation in parental MCF7 (MCF7/P) and MCF7/TAM cells treated with indicated concentrations of Broussoflavonol B (BB) for 12 hours. B. Cells were treated with indicated concentrations of tamoxifen (TAM) together with vehicle or 1 µM of Broussoflavonol B (BB) for seven days and the numbers of survived cells were counted. The columns represent the means of three experiments; bars, SE. *, P<0.05 for MCF/TAM cells treated with vehicle vs cells treated with 1 µM of tamoxifen and BB.

We then examined whether the ER-α36 downregulator Broussoflavonol B (BB) [32], [33] is also able to disrupt the ER-α36-EGFR/HER2 loops and restores TAM sensitivity in TAM-resistant cells. We treated MCF7/TAM cells with different concentrations of BB for 12 hours and the steady state levels of ER-α36, EGFR and HER2 were examined with Western blot analysis. We found that BB potently down-regulated ER-α36 expression and phosphorylation levels of EGFR and HER2 but weakly down-regulated expression levels of EGFR and HER2 proteins in MCF7/TAM cells (Figure 5A). BB treatment also significantly sensitized MCF7/TAM cells to TAM (Figure 5B). When we increased BB treatment to 36 hours, we observed the expression levels of EGFR and HER2 were dramatically downregulated in the cells treated with BB for 36 hours (Figure 4C). Our results thus demonstrated that ER-α36 downregulator BB was also able to disrupt the ER-α36-EGFR/HER2 positive regulatory loops and restored TAM sensitivity.

### ER-positive breast cancer stem/progenitor cells express ER-α36-EGFR/HER2 positive regulatory loops

**Figure 6:**
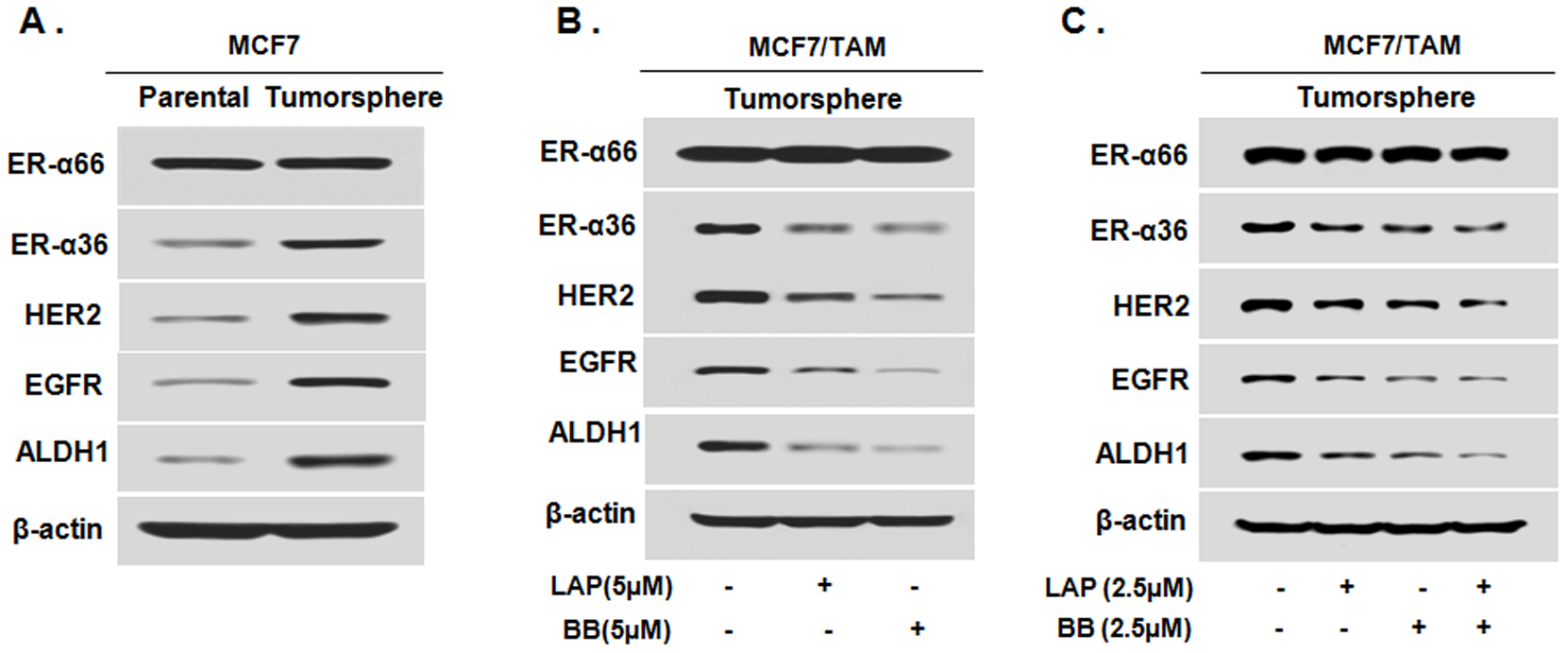
Tumorsphere cells derived from ER-positive breast cancer MCF7 cells express ER-α36-EGFR/HER2 positive regulatory loops. A. Western blot analysis of the expression of ER-α36, ER-α66, EGFR, HER2 and ALDH1 in the monolayer MCF7 cells grown on attachment dishes (MCF7/P) and MCF7 tumorsphere cells grown on low-attachment dishes. B. Tumorsphere cells derived from MCF7TAM cells were treated with 5 µM of Broussoflavonol B (BB) or Lapatinib (LAP) for five days. Western blot analysis of expression levels of different proteins was performed. C. Tumorsphere cells derived from MCF7TAM cells were treated with 2.5 µM of Broussoflavonol B (BB), Lapatinib (LAP) or BB (2.5 µM) and LAP (2.5 µM) together for five days. Western blot analysis of expression levels of different proteins was performed.

Recently, we found that ER-positive breast cancer stem/progenitor cells express higher levels of ER-α36 [33]. We sought to investigate whether there exist ER-α36-EGFR/HER2 regulatory loops in ER-positive breast cancer stem/progenitor cells and disruption of these loops sensitizes these cancer stem/progenitor cells to TAM. We cultured ER-positive breast cancer MCF7 cells in the tumorsphere media and under suspension condition to form tumorspheres, and performed Western blot analysis to assess expression of ER-α36, EGFR and HER2 in tumorsphere and parental cells. We found that ER-α36, EGFR and HER2 were all highly expressed in tumorsphere cells compared to parental cells (Figure 6A). ALDH1, a functional marker of breast cancer stem/progenitor cell [34]–[36], was also highly expressed in tumorsphere cells (Figure 6A). The results thus demonstrated that there exist ER-α36-EGFR/HER2 regulatory loops in ER-positive breast cancer stem/progenitor cells.

We then sought to examine whether Lapatinib and Broussoflavonol B are still able to disrupt the ER-α36-EGFR/HER2 regulatory loops in ER-positive breast cancer stem/progenitor cells. We cultured MCF7/TAM cells under suspension conditions to form tumorspheres for five days and then Lapatinib (LAP, 5 µM) or Broussoflavonol B (BB, 5 µM) was added for five days. Western blot analysis revealed that both Lapatinib and Broussoflavonol B were able to disrupt the ER-α36-EGFR/HER2 regulatory loops in the breast cancer stem/progenitor cells derived from MCF7/TAM cells (Figure 6B). We also tested the effects of combination of both Lapatinib and Broussoflavonol B on the ER-α36-EGFR/HER2 regulatory loops. We found that the combination treatment also effectively disrupted the ER-α36-EGFR/HER2 regulatory loops (Figure 6C) but failed to observe any synergistic effects of two chemicals.

### Disruption of ER-α36-EGFR/HER2 positive regulatory loops sensitizes ER-positive breast cancer stem/progenitor cells to TAM

**Figure 7:**
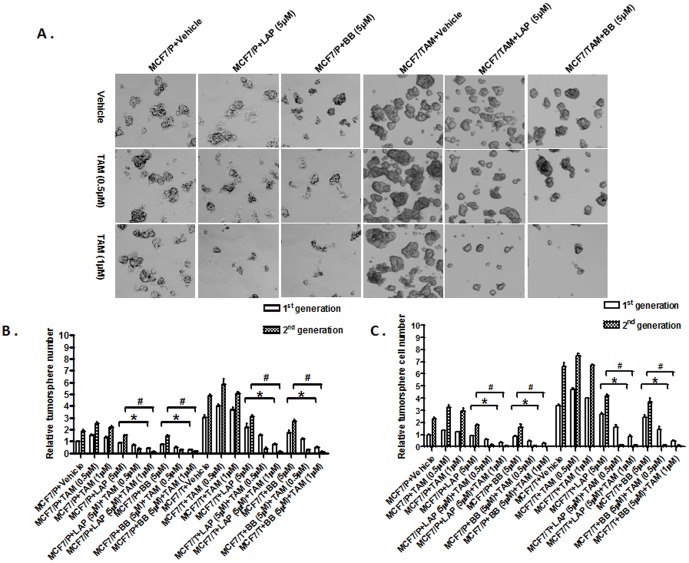
Disruption of the ER-α36-EGFR/HER2 positive regulatory loops sensitizes ER-positive breast cancer stem/progenitor cells to TAM. A. Tumorsphere formation assay was used to assess the effects of TAM alone or together with Lapatinib (LAP) or Broussoflavonol B (BB) on ER-positive breast cancer stem/progenitor cells derived from parental MCF7 (MCF7/P) and tamoxifen resistant MCF7 cells (MCF7/TAM). The representative results are shown. B. The numbers of tumorspheres formed by MCF7/P and MCF7/TAM cells in the presence or absence of LAP and BB for 1^st^ and 2^nd^ generations. C. The number of cells from dissociated tumorspheres formed by MCF7/P and MCF7/TAM cells in the presence or absence of LAP and BB for 1^st^ and 2^nd^ generations. The columns represent the means of three experiments; bars, SE. * & #, P<0.05 for MCF/TAM cells treated with vehicle vs cells treated with tamoxifen and LAP or BB, respectively.

We then sought to examine whether disruption of the ER-α36-EGFR/HER2 positive regulatory loops will also sensitize ER-positive breast cancer stem/progenitor cells to TAM. We cultured MCF7 cells under suspension conditions to form tumorspheres for five days and then different concentrations of TAM together with Lapatinib (LAP, 5 µM) or Broussoflavonol B (BB, 5 µM) were added for another five days. We found that in the presence of Lapatinib or Broussoflavonol B, ER-positive breast cancer stem/progenitor cells of the tumorspheres formed by MCF7 and MCF7/TAM cells became sensitive to TAM; TAM reduced the number of tumorspheres (Figure 7A & B). We also dissociated cells from tumorspheres and examined cell number of tumorspheres, and found the cell number from the tumorspheres formed by both MCF7 and MCF7/TAM was significantly reduced in the presence of TAM together with Lapatinib or Broussoflavonol B (Figure 7C) while TAM alone enhanced cell growth at lower concentrations in the tumorsphere cells derived from MCF7 and MCF7/TAM cells (Figure 7C).

**Figure 8:**
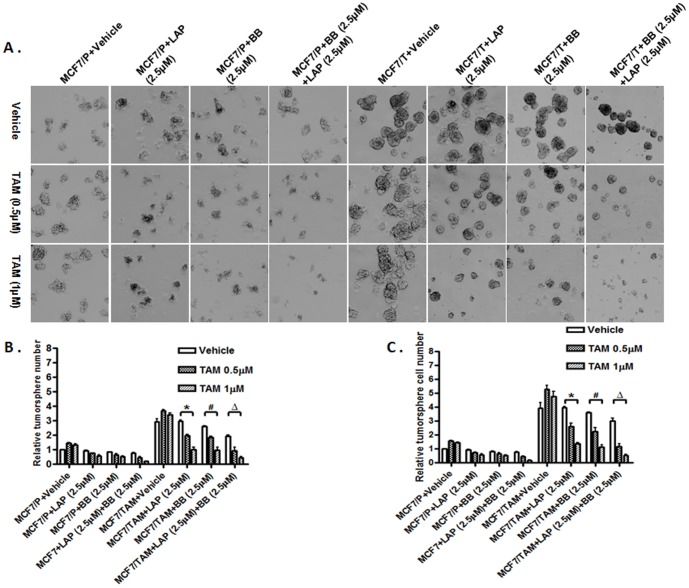
Combination of Broussoflavonol B and Lapatinib sensitizes ER-positive breast cancer stem/progenitor cells to TAM. A. Tumorsphere formation assay was used to assess the effects of TAM alone or together with 2.5 µM of Lapatinib (LAP), Broussoflavonol B (BB) or BB (2.5 µM) and LAP (2.5 µM) on ER-positive breast cancer stem/progenitor cells derived from parental MCF7 (MCF7/P) and tamoxifen resistant MCF7 cells (MCF7/TAM). The representative results are shown. B. The numbers of tumorspheres formed by MCF7/P and MCF7/TAM cells in the absence or presence of LAP (2.5 µM), BB (2.5 µM) or LAP (2.5 µM) and BB (2.5 µM) together. C. The number of cells from dissociated tumorspheres formed by MCF7/P and MCF7/TAM cells in the absence or presence of LAP (2.5 µM), BB (2.5 µM) or LAP (2.5 µM) and BB (2.5 µM) together. The columns represent the means of three experiments; bars, SE. *, # and Δ, P<0.05 for MCF/TAM cells treated with vehicle vs cells treated with tamoxifen and LAP, BB, or LAP+BB together.

To assess the effects of disruption of the positive–regulatory loops on the self-renewal of the stem-like cells, we also studied the tumorsphere formation of MCF7 and MCF7/TAM cells as well as their derivatives with different levels of ER-α36 expression through serial passages in the absence or presence of Lapatinib or Broussoflavonol B. The cells were treated with vehicle or chemicals at the time of the sub-seeding. We found that combination of TAM and Lapatinib or Broussoflavonol B dramatically inhibited tumorsphere number in the second generation of tumorspheres (Figure 7 B & C), suggesting that disruption of the positive-regulatory loops also inhibits the self-renewal of the breast cancer stem/progenitor cells. Finally, we tested the effects of combination of both Lapatinib and Broussoflavonol B together with TAM on the growth of stem/progenitor cells derived from MCF7 and MCF7/TAM cells. We found that the combination of two chemicals effectively inhibited tumorsphere formation (Figure 8). Again, we did not observe any synergistic effect.

## Discussion

TAM therapy is the most effective treatment for advanced ER-positive breast cancer, but its effectiveness is limited by high rate of resistance acquired during treatment. Previously, we reported that breast cancer patients with tumors expressing high levels of endogenous ER-α36 less benefited from TAM therapy than those with low levels of ER-α36 expression [23], suggesting elevated expression of ER-α36 is a mechanism underlying acquired tamoxifen resistance. Recently, others and we confirmed that elevated ER-α36 expression is involved in TAM resistance through mediating agonist activity of TAM [26], [27].

Here, we showed that TAM induced expression of ER-α36, EGFR and HER2 in TAM sensitive MCF7, T47D and H3396 cells. In addition, MCF7/TAM cells selected by long-term cultivation in the presence of TAM also expressed elevated levels of endogenous ER-α36, EGFR and HER2, which results in more rapid cell growth compared to the parental MCF7 cells. Knockdown of ER-α36 expression in MCF7/TAM cells reduced EGFR and HER2 expression. We also showed that MCF7 cells with forced expression of ER-α36 expressed increased levels of EGFR and HER2. Taken together, our results indicated that the ER-α36-EGFR/HER2 positive regulatory loops are one of the underlying mechanisms of ER-positive breast cancer cells gained expression of the growth factor receptors during TAM treatment.

Previously, we found that antiestrogens TAM and ICI 182, 780 failed to block ER-α36-mediated non-genomic estrogen signaling [21]. Recently, we also found that ER-α36 mediated agonist activities of both TAM and ICI 182, 780 in ER-negative breast cancer cells that express high levels of endogenous ER-α36 [24] and elevated ER-α36 expression is one of the underlying mechanisms of TAM resistance [26], [27]. Here we showed that MCF7/TAM cells exhibited a biphasic growth curve in response to TAM; increasing cell growth at low concentrations and failed to do so at higher concentrations, consistent with previous findings that some breast cancers may be initially growth inhibited by TAM, and later become dependent on TAM for proliferation [5], [6]. It worth noting that the TAM at higher concentrations was still able to inhibit growth of MCF7/TAM cells, suggesting that TAM resistance is a concentration dependent event; high concentrations of TAM still exhibit cytotoxic activity in cells insensitive to low concentrations of TAM.

In the current study, we observed that TAM induced ER-α36, EGFR and HER2 expression through the positive regulatory loops and inhibition of both EGFR and HER2 signaling pathways with the dual kinase inhibitor Lapatinib disrupted the regulatory loops and restored TAM sensitivity. Our results thus are in good agreement with the previous reports that Lapatinib restores antiestrogen sensitivity in breast cancer cells with acquired endocrine resistance [19], [37]. Here, we observed that Lapatinib inhibited phosphorylation of both EGFR and HER2 and downregulated ER-α36. Our data thus provided a novel molecular mechanism to the function of the dual kinase inhibitor Lapatinib; disruption of the ER-α36-EGFR/HER2 positive regulatory loops. It is worth noting that it has been reported that Lapatinib at 1 µM modestly induced HER2 expression in ER-negative breast cancer SKBR3 and MCF7-HER2 cells that over-express HER2 [38], which seems contradictory to our findings. We performed experiments with different concentrations of Lapatinib in MCF7/HER2-18 cells that were stably transfected with a HER2 expression vector [39] and found that Lapatinib at 100 nM and 1 µM indeed modestly increased HER2 expression while higher concentrations of Lapatinib (>1 µM) decreased the steady state level of HER2 (data not shown). Thus, Lapatinib regulation of HER2 expression may be a concentration dependent event.

Previously, we found that the potent ER-α disruptor ICI 182, 780 failed to degrade ER-α36 due to the lacking of the critical Helix 12 in the C-terminal of ER-α36 protein [40]. Recently, we found that a falconoid, Broussoflavonol B (5, 7, 3', 4'-Tetrahydroxy-3-methoxy-6,8-diprenylflavone) purified from the bark of Broussonetia papyrifera was able to downregulate ER-α36 expression and to inhibit proliferation of ER-negative breast cancer cells [32], [33]. Here, we showed that Broussoflavonol B was also able to disrupt the ER-α36-EGFR/HER2 positive regulatory loops and restored TAM sensitivity in TAM resistant cells. We also showed that Broussoflavonol B treatment was able to block the induction of the positive regulatory loops by TAM in MCF7 cells. Thus, further development of chemical compounds like Broussoflavonol B may provide novel approaches to restore TAM sensitivity in TAM resistant cells or to block the development of acquired TAM resistance.

Accumulating experimental and clinical evidence indicate that breast cancer arises from mammary stem/progenitor cell populations [41]–[43]. Although the possible involvement of breast cancer stem/progenitor cells in TAM resistance has been proposed [44] and demonstrated [45], the exact function and the underlying mechanism of breast cancer stem/progenitor cells in TAM resistance remain largely unknown. Many signaling pathways involved in regulation of normal mammary stem cells including Hedgehog, Bmi-1, Wnt, NOTCH, HER2, p53 and PTEN/Akt/β-catenin pathways play roles in breast cancer stem/progenitor cells [46]–[49]. Recently, we reported that ER-positive breast cancer stem/progenitor cells express higher levels of ER-α36 [34]. Here, we showed that stem-like cells in the tumorspheres derived from MCF7 express elevated levels of ER-α36, EGFR and HER2, indicating there exist the ER-α36-EGFR/HER2 regulatory loops in the ER-positive breast cancer stem/progenitor cells. Again, disruption of these regulatory loops with Lapatinib or Broussoflavonol B was able to sensitize ER-positive breast cancer stem/progenitor cells to TAM. Our results thus provided rationales to develop novel therapeutic approaches to treat breast cancer via eliminating breast cancer stem/progenitor cells by targeting the ER-α36-EGFR/HER2 loops.

In this study, we also tested the effects of combination of both Lapatinib and Broussoflavonol B on the ER-α36-EGFR/HER2 regulatory loops and on the growth of tumorsphere cells derived from MCF7/TAM cells. We found that the combinational treatment effectively disrupted the ER-α36-EGFR/HER2 regulatory loops and inhibited growth of tumorsphere cells. However, we did not observe any synergistic effects of two chemicals, which is in good agreement with our hypothesis that both Lapatinib and Broussoflavonol B affect the same ER-α36-EGFR/HER2 regulatory loops.

In summary, here we provided evidence to demonstrate the existence of ER-α36-EGFR/HER2 positive regulatory loops in TAM resistant breast cancer cells and that disruption of these regulatory loops restored TAM sensitivity in these cells. Our findings that elevated expression of the ER-α36-EGFR/HER2 regulatory loops is one of the mechanisms by which ER-positive breast cancer cells escape the hormonal therapy based on estrogen deprivation provided a rational to develop novel therapeutic approaches for TAM resistant patients by targeting these regulatory loops.
